# ASSOCIATION BETWEEN WEIGHT-TEASING AND PHYSICAL ACTIVITY IN ADOLESCENTS

**DOI:** 10.1590/1984-0462/;2017;35;3;00005

**Published:** 2017-07-31

**Authors:** Priscila Iumi Watanabe, Fabio Eduardo Fontana, Michael Pereira da Silva, Oldemar Mazzardo, Eliane Denise Araújo Bacil, Wagner de Campos

**Affiliations:** a Universidade Federal do Paraná, Curitiba, PR, Brasil.; b University of Northern Iowa, Cedar Falls, IA, Estados Unidos.; c Universidade Estadual do Oeste do Paraná, Marechal Cândido Rondon, PR, Brasil.

**Keywords:** Physical activity, Adolescent, Teasing

## Abstract

**Objective:**

The aim of the study was to determine the association between weight-teasing and physical activity in students from public schools of Curitiba, Paraná (Southern Brazil).

**Methods:**

The sample consisted of 95 students (48 boys and 47 girls) aged 12 to 14 years old. The Perception of Weight Teasing (POTS) and The Perception of Weight Teasing during Physical Activity Scale assessed the frequency of weight-teasing experienced by the participants. Accelerometers measured physical activity. BMI assessed the weight status of the participants. Pearson correlations analyzed the association between the teasing and physical activity variables at a significance level of 0.05.

**Results:**

The relationship between teasing variables and physical activity was not significant. A large proportion of participants failed to meet the recommended levels of physical activity regardless of sex (72%), and girls were significantly less physically active than boys (56.3% of boys and 89.4% of girls; p<0.01).

**Conclusions:**

Some participants were targets of weight-teasing, but teasing was not related to physical activity. Interventions are necessary to educate middle school students about the harmful consequences of weight teasing.

## RESUMO

**Objetivo:**

Investigar a associação entre a atividade física e a provocação referente ao peso corporal em escolares matriculados no sistema público de ensino em Curitiba, Paraná.

**Métodos:**

A amostra foi composta por 95 escolares (48 meninos e 47 meninas) com idades entre 12 e 14 anos. Para avaliar a provocação, foram utilizadas as versões em português da Escala de Percepção de Provocação e a Escala de Percepção de Provocação Durante a Atividade Física. A atividade física foi mensurada por meio de acelerômetros. O Índice de Massa Corporal (IMC) foi utilizado para definição do estado nutricional dos participantes. A associação entre as variáveis de provocação e de atividade física foi analisada por meio da correlação de Pearson, adotando um nível de significância de 0,05.

**Resultados:**

Não foram verificadas associações significativas entre as variáveis de provocação e atividade física. Foram observados valores elevados de indivíduos insuficientemente ativos (72%), com diferenças significativas entre os sexos (56,3% dos meninos e 89,4% das meninas, *p*<0,01).

**Conclusões:**

A provocação referente ao peso é vivenciada pelos escolares avaliados e não esteve relacionada com a atividade física. Destaca-se a importância de intervenções que incentivem a conscientização das consequências da provocação referente ao peso.

## INTRODUCTION

Weight-teasing is a common form of bullying, defined as negative communication from an agent regarding the weight of a target person, in which elements of humor, aggressiveness and ambiguity are present.^1^ ^,^ ^2^ It can be expressed through name calling, insinuations, social exclusion, imitations, among others.^3^ ^,^ ^4^ Weight-teasing is perceived more frequently by overweight and female individuals.^6^ ^,^ ^7^ Evidence indicates that provocation can affect the psychological well-being of the targets,^6^ ^,^ ^8^ ^,^ ^9^ besides being associated with the acquisition of behaviors harmful to health, such as eating disorders and reduced levels of physical activity.^7^ ^,^ ^10^ ^,^ ^11^ ^,^ ^12^

Greenleaf et al.^13^ suggest that teenagers who are targets of teasing present lower levels of self-concept and less self-efficacy to practice physical activity. In addition, weight-teasing that occurs in the context of physical activity practice was inversely related to pleasure in the practice of sports and low-intensity physical activity in leisure time.^7^ These findings suggest there is a tendency for victims of weight-teasing to prefer sedentary/isolated activities rather than active/social activities, and also stress the importance of conducting research on this construct among Brazilian adolescents.^14^

To date, there is a single study published with a Brazilian sample regarding the perception of weight teasing and its association with psychological and behavioral outcomes in adolescents. Leme and Philippi^15^ suggest that Brazilian adolescents who reported being subjected to weight teasing were more likely to present unhealthy behaviors to control weight. The present study is pioneer in approaching the association of this form of bullying with the practice of physical activity, a relevant theme considering the importance of physical activity for health maintenance,^16^ especially among overweight and female individuals, who, aside from being more prone to provocation related to body weight,^6^ also seem to present lower levels of physical activity.^17^

This study aimed at investigating the association between weight teasing, in general and during the practice of physical activity, with the variable physical activity in schoolchildren from the city of Curitiba (PR).

## METHOD

The nonprobability sample consisted of adolescents of both sexes, aged between 12 and 14 years old, enrolled in the public school system of the city of Curitiba, Paraná. The schools were selected intentionally and all students in the classes that belonged to the predetermined age group were invited. A total of 146 students participated in the study; however, only 95 (48 boys and 47 girls) presented valid accelerometer data, characterizing a sample loss of approximately 34%. The power of the final sample was calculated afterwards with the G*Power software, version 3.1, considering a 5% significance level. The results indicated 80% power to detect correlations with significance equal to or higher than 0.26.

The economic class was determined using the Brazil Economic Classification Criteria (CCEB), which accounts for items in the respondent’s household, as well as information about the head of the household’s schooling and the presence of public services (piped water and paved street).^18^ For the analyses, strata A1 and A2 were grouped in A, strata B1 and B2 in B and strata C1 and C2 in C. There were no students classified in classes D and E.

Subject height and body mass were evaluated according to the procedures described by Tritschler for further calculation of body mass index (BMI).^19^ Cut-off points for this variable followed the criteria proposed by Cole et al.^20^ In the present study, participants who were overweight or obese were classified in the “Overweight” group.

General weight teasing was evaluated by the Perception of Teasing Scale, which corresponds to the translated and transculturally adapted version of the Perception of Teasing Scale (POTS) for Brazilian adolescents. This questionnaire was transculturally adapted and validated for Brazilian adolescents with a rigorous process consisting of the following stages:

1. Translation.
2. Reverse translation.
3. Review by a committee.
4. Content validity.
5. Internal consistency.
6. Good Internal Consistency (Cronbach’s α=0.87) and Reproducibility test-retest (ICC=0.98, CI=0.97-0.98).

This version is composed of five questions:

1. People made fun of you because you were overweight.
2. People made jokes about you being too heavy.
3. People laughed at you when you tried to participate in sports because you were overweight.
4. People called you names, such as “fat”.
5. People pointed out to you because you were overweight.

These questions address the frequency with which participants have experienced such teasing on a likert-type scale ranging from one (never) to five (very frequent) and are followed by the question “If this happened, how upset were you?”, ranging from one (not upset) to five (very upset). The present study considered teasing in a continuous manner analyzing the value of the score generated by the sum of the questions - in which high scores represent greater history of teasing and higher emotional impact - and in a dichotomous manner, classifying the report as teasing if the participant answered any question with something other than never.

The construct of weight teasing during physical activity was estimated through the test of Perception of Weight Teasing During Physical Activity, a version adapted for Brazilian adolescents from the Weight Criticism During Physical Activity,^7^ which underwent the same validation process as the Perception of Teasing Scale and also presents good internal consistency values (Cronbach’s α=0.86) and test-retest reproducibility (ICC=0.96, CI=0.94-0.99). The version in Portuguese of this instrument is composed of four questions that address the frequency of teasing:

1. People made fun of you when you did sports or exercises because you were overweight.
2. People made jokes about you being very heavy during the practice of sports or physical exercises.
3. People have insulted you with name-calling related to you being heavy when you did sports or exercise.
4. People looked at you with contempt when you wore sportswear for exercise or sports because you were overweight.

These questions are followed by the question “If this happened, how upset were you?”. The classification (continuous and dichotomous) followed the format used for the Perception of Teasing Scale.

Physical Activity was evaluated using the ActiGraph GT3X accelerometers (ActiGraph; Pensacola, FL) programmed to collect data and summarize them in 60-second epochs. From these data, physical activity was stipulated in a continuous way, in minutes per day spent doing moderate to vigorous physical activity, or moderate to vigorous physical activity (MVPA), by the algorithm developed by Trost et al.^21^ Students were instructed to place the accelerometer on their hip, aligned with the middle axillary line, beginning the use of the devices in the morning and ending at night. This procedure was performed for seven consecutive days. The day was considered valid if it had at least 480 minutes of use. The valid week of use corresponded to at least four valid monitoring days, including at least one weekend day.^22^ Weekly physical activity was measured by multiplying the daily average of physical activity by seven. Physical activity was also expressed in a dichotomous manner, classifying adolescents as sufficiently active (≥300 minutes MVPA per week) or insufficiently active (<300 MVPA per week).^23^

Data collection was performed between September and November 2015, by the main researcher and a trained team from the Center for Physical Activity and Health Studies at Universidade Federal do Paraná (CEAFS-UFPR). The self-administered questionnaires (Perception of Teasing Scale, Weight Teasing During Physical Activity and the Economic Classification Questionnaire)^7^ ^,^ ^11^ ^,^ ^18^ were applied in a reserved room. Likewise, the anthropometric measures (body mass and height) were taken in a reserved place. At the end of the collection, accelerometers were delivered to be used for one week.

All participants submitted the Informed Consent signed by the parents or guardians, authorizing their participation, and the Term of Assent (TA) duly signed by the participants. This research was approved by the Human Research Ethics Committee/UFPR, protocol number CAAE 39266214.3.0000.0102.

Mean and standard deviation were used to characterize and describe the sample. Prevalence was described by the distribution of simple and relative frequencies. Normality was verified by the Kolmogorov Smirnov test and by the Skewness analysis, indicating non-parametric distribution of the data. Logarithmic (physical activity) and square root (teasing variables) transformations were used to normalize the data. The chi-square test was used to compare proportions. In order to analyze the association between weight teasing and teasing during physical activity (TDPA) with the variables of physical activity, the Pearson correlation was also adopted. The analyses were carried out using the Statistical Package for Social Sciences (SPSS), version 21.0, with a 5% significance level.

## RESULTS

The final sample consisted of 95 adolescents with a mean decimal age of 13.2±0.8 years, with boys aged 13.4 ± 0.8 years old and girls aged 12.9 ± 0.8 years old. Table 1 shows the distribution of participants according to the study variables, as well as the results of the comparisons between both genders. The prevalence of overweight was a matter of concern, as well as insufficiently active participants. In addition, it is noted that teasing and TDPA are experienced by the adolescents evaluated. The results of the comparison between both genders showed significant differences only regarding the physical activity variable (*p*<0.01), indicating that female adolescents were more insufficiently active.

**Table 1:**
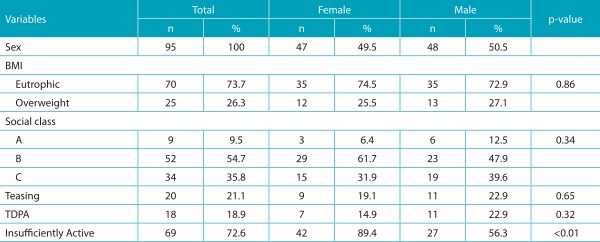
Descriptive data and sex differences.

Results in absolute and relative frequency; BMI: body mass index; TDPA: teasing during physical activity.

Table 2 presents the prevalence of weight-teasing in general and during physical activity in the sample divided by gender and nutritional status. The results indicate the existence of significant differences between the groups with normal weight and overweight. The overweight group had higher prevalence of weight-teasing and TDPA for the total sample and for males.

**Table 2:**
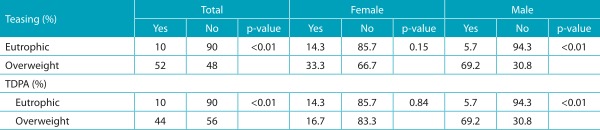
Comparisons of proportions of adolescents who reported-weight teasing and TDPA according to body mass index (eutrophic and overweight).

BMI: body mass index; TDPA: teasing during physical activity.

Regarding the associations, no significant correlations were found between the continuous variables of physical activity and the variables of weight-teasing in both sexes (Table 3). The association between weight-teasing and MVPA was weak and non-significant (r=0.05, *p*=0.60), and the same was observed for weight-teasing during physical activity (r=0.03, *p*=0.76).

**Table 3:**
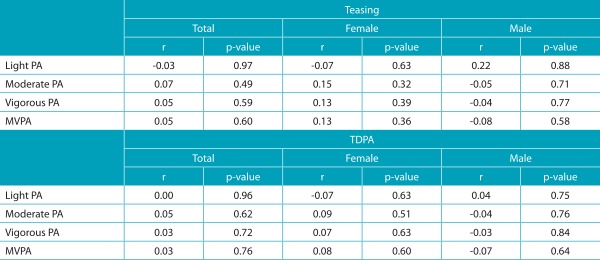
Correlation between physical activity and weight-teasing and TDPA.

TDPA: teasing during physical activity; PA: physical activity; MVPA: moderate to vigorous physical activity; r (rho): Pearson’s correlation coefficient.

## DISCUSSION

According to the results, there were no significant associations between teasing and TDPA with physical activity. In the statistical context, it is likely that the high percentage of insufficiently active participants in the sample made it impossible to verify significant relationships between the variables. However, this may also be due to the dependence of the presence of other factors in order for the teasing to influence physical activity, as demonstrated in the study by Jensen and Steele,^12^ in which there was an inverse relationship between TDPA and vigorous physical activity only among girls with high body dissatisfaction. In addition, it is possible that, for this age group, teasing is related to other behaviors and not to physical activity specifically, such as risk behaviors for weight loss and aspects related to psychological well-being. Finally, it is still important to consider that, for some individuals in the present sample, provocation has a positive effect on the practice of physical activity, acting as a motivating factor.

Despite the lack of studies involving Brazilian adolescents, research in the United States shows evidence that weight-related provocation has a negative impact on physical activity, and may influence adolescents to choose sedentary/isolated activities rather than active/social ones.^14^ In 2014, a study with 1,419 schoolchildren found that weight-teasing may affect several factors related to physical activity, as the victims of teasing presented with lower levels of physical self-concept regarding their aerobic capacity, strength and flexibility, as well as lower self-efficacy for the practice of physical activity.^13^ As for TDPA, Faith et al. found inverse and moderate associations between the coping skills of the participants and the pleasure of practicing sports, perceived physical activity compared to peers and low-intensity activity during leisure time.^7^ Possibly, the different results comparing these studies and the present study stem from cultural differences and the instruments used to evaluate physical activity, which contrasts with the objectivity of the accelerometer.

The literature has shown that, in addition to presenting greater risks for comorbidities,^24^ ^,^ ^25^ overweight individuals suffer more frequent teasing, for a longer period of time (in years), and report higher emotional impact because of them. The prevalence of weight-teasing in Canadian adolescents was 22% for those with normal weight and 45% for those who were overweight.^6^ This study showed that 10% of the group classified as eutrophic reported teasing against 52% of the overweight group. About the teasing that occurred specifically in the context of physical activity (TDPA), the values found were 10% for eutrophic and 44% for overweight participants. Such difference was also seen among boys. However, this did not occur for the females in the present sample. This is probably because a considerable number of girls classified as eutrophic also suffer from teasing. Possibly, they were considered overweight by colleagues, even at normal weight, due to the thinness ideal imposed by the current culture.^25^

Goldfield et al. have shown that the prevalence of weight-teasing occurs more frequently among girls than boys, but this was not observed in the present study.^6^ This may be due to the increasing social pressure for male individuals to adhere to aesthetic standards that consider an ideal body to be that lean or muscular body.^25^ Thus, boys would be targets of teasing regarding their weight just as much as girls.

These teasing episodes are worrisome, since evidence suggests that being a victim of weight-teasing can cause harmful consequences for the psychological well-being, such as low self-esteem, depressive symptoms and body dissatisfaction.^8^ ^,^ ^13^ In addition, such stigmas are also related to the acquisition of risk behaviors, such as inadequate attitudes for body weight control (use of laxatives, induction of vomiting and dieting)^15^ and represent a limitation to the practice of physical activity.^26^

Considering that physical activity is an important factor to prevent the decline of physical and mental health,^27^ it is important to investigate the relationship between the reduction of physical activity in the young population and the weight-related teasing, even if the signs of this relationship are inconsistent. This is important, especially when studies have shown that, despite the efforts to promote an active lifestyle, a large proportion of the young population is still not active enough. This can be observed in data from 105 countries, in which 80.3% (CI 80.1-80.5) of adolescents, aged between 13 and 15 years old, do not comply with recommendations for physical activity.^17^ In Curitiba, these values are also alarming, being 78.4% for boys and 90.4% for girls, as found in a representative study with schoolchildren.^28^ Likewise, for our sample, the proportion of underactivity remains high, and even higher among girls (56.3% for boys and 89.4% for girls).

Despite the sample limitations, due to the number of participants that made it impossible to use the analyses with the control of intervening variables, it is worth noting the relevance of this study, since it is the first to investigate these variables in Brazil. In addition, this work contributes for being the first investigation, in this subject, to measure physical activity in an objective manner, using the accelerometer. Further research on the subject is still required in Brazil. It is suggested that future studies investigate the relationship between teasing and physical activity, considering eating behaviors as well as the moderating effect of psychosocial aspects in this relationship.

The results of the present study demonstrated that weight-teasing and TDPA are also experienced by adolescents from Curitiba, Paraná. Unlike previous studies, no significant differences were observed between the proportions of boys and girls suffering from weight-teasing and TDPA, indicating that this is a problem that affects both sexes. There is still high prevalence of insufficiently active adolescents, especially among females. Thus, it is important to emphasize the importance of interventions aimed at raising awareness for the harmful aspects of weight-related provocation. It is also worth noting the need for further research on the subject to understand the relationship between physical activity and weight-teasing in Brazilian adolescents.
